# Preparation of Cellulose Nanocrystals by Synergistic Action of Ionic Liquid and Recyclable Solid Acid under Mild Conditions

**DOI:** 10.3390/molecules28073070

**Published:** 2023-03-30

**Authors:** Li Ma, Yongjian Xu, Jian Chen, Cuihua Dong, Zhiqiang Pang

**Affiliations:** 1 College of Bioresources Chemical and Materials Engineering, National Demonstration Center for Experimental Light Chemistry Engineering Education, Shaanxi University of Science & Technology, Xi’an 710021, China; 2 Faculty of Light Industry, Qilu University of Technology (Shandong Academy of Science), Jinan 250353, China; 3 Shandong Jincailun Paper Industry Co., Ltd., Liaocheng 252300, China

**Keywords:** cellulose nanocrystals, ionic liquids, solid acid, swelling, hydrolysis, recycling

## Abstract

Cellulose nanocrystals (CNCs) are nanoscale particles made from cellulose. They have many unique properties such as being lightweight, stiff, and renewable, making them promising for a variety of applications in a wide range of industries, including materials science, energy storage, and biomedicine. In this paper, a two-stage (swelling-SA-catalyzed) method including IL pretreatment and solid acid hydrolysis process was developed to extract CNCs with high purity and good thermal stability from microcrystalline cellulose (MCC). In the first stage, the swelling of MCC in ionic liquid was studied with the assistance of ultrasonication, and it was found that the amorphous regions became more disordered while the crystalline areas were selectively retained under the conditions of 30 min of reaction time, 45 °C of temperature, 2% of ionic liquid water content and 1:4 mass ratio of cellulose to ionic liquid. CNCs were extracted using solid acid hydrolysis, with a 45 wt% solid acid to cellulose ratio and a 5.0 h hydrolysis process at 45 °C. The morphology, crystallinity, surface characteristics and thermo stability of the sample were characterized by atomic force microscopy (AFM), X-ray diffraction (XRD) and thermogravimetric analysis (TGA), respectively. Results demonstrated the highly thermostable CNCs were successful extracted with rodlike shape of 300 ± 100 nm in length and 20 ± 10 nm in width. Solid acid recovery and reuse were also studied, revealing a promising candidate that can reduce the environmental impact associated with chemical products.

## 1. Introduction

Cellulose, the major component of plants, is a renewable natural resource present in the cell wall in the form of microfibrils and is one of the most abundant renewable natural resources on earth [1]. With its ordered crystalline structure and random amorphous structure, it offers great potential as a renewable and sustainable alternative to petroleum [2,3]. Cellulose nanocrystals (CNCs) is a significant product of cellulose, featuring high surface area, high specific strength, crystallinity, low density, biodegradability, nontoxicity and biocompatibility. Typically, CNCs is a rodlike cellulose whisker with a diameter range of 5–70 nm and lengths of hundreds of nanometers [4]. The size of CNCs is highly dependent on the original material and the preparation methods and conditions employed. Due to its unique properties, CNCs has demonstrated promising applications in multiple areas, including polymer reinforcement, nanocomposites, electronics, batteries, drug delivery, energy, and environmental engineering [5,6,7].

Isolation of cellulosic crystalline whiskers typically requires a multistep process involving chemical and/or physical procedures [8]. The amorphous phase of cellulose, which is formed by disruption of hydrogen bonds, is relatively easy to break, while the nanostructured crystalline phase, held together by van der Waals forces and strong inter- and intramolecular hydrogen bonding, is much more difficult to break [9]. The key to preparing CNCs is to maximize the removal of the amorphous region while preserving the crystalline region intact. Various techniques have been utilized to extract CNCs from lignocellulosic materials, including acid hydrolysis, oxidation method, mechanical treatment, enzymatic hydrolysis, ionic liquids treatment, and a combination of multiple methods [10]. The commonly used method of acid hydrolysis, mainly involving the use of concentrated sulfuric acid or hydrochloric acid, has been observed to yield relatively high levels of CNCs compared with other methods. However, this method is accompanied by the disadvantages of requiring large dosages of acids, high water consumption, and low thermostability of the products [11,12]. A 65% (*w*/*w*) sulfuric acid solution has been widely used to hydrolyze cellulosic materials under heating conditions ranging from 45–60 °C, as this could effectively remove amorphous regions while preserving crystalline regions to a great extent. However, sulfate groups formed on the surface of CNCs during hydrolysis have a detrimental effect on the thermal stability and accelerate the degradation of products [13,14,15]. Additionally, the strong acid can also corrode the production equipment, which is a major challenge in industrial production.

TEMPO-mediated oxidation has been utilized to improve the aqueous solubility of CNCs by introducing carboxylate groups onto their surface. The grafting of polymers onto native celluloses has been shown to be an effective approach for surface modification [16]; however, it also leads to decreased thermal stability and difficulty in recovering toxic compounds [17]. The downside of using a combination of mechanical and chemical methods for CNCs extraction is the high energy consumption required [18,19]. Enzymatic hydrolysis using cellulose enzymes can be employed as a pretreatment method to facilitate the delamination of lignocellulose into CNCs under milder conditions by targeting the amorphous regions [20,21,22,23]. The energy demand has decreased when enzymatic pretreatment is applied prior to the mechanical treatments; however, a long enzymolysis period is needed [24]. Exploring CNCs extraction processes that are environmentally friendly and cost-effective, reducing consumption of acid, energy, and other toxic compounds, and achieving high purity and thermostability of CNCs is highly desirable. 

Ionic liquids (ILs) have the advantages of being chemically stable and having relatively low melting temperatures, making them suitable for use as novel solvents with the ability to swell, dissolve, or hydrolyze under certain conditions [25]. Ionic liquids have been increasingly studied as cost-effective and ecofriendly solvents due to their wide liquid range, excellent solubility, zero vapor pressure, and potential for reuse. Cellulose was dissolute in 1-allyl-3-methylimidazolium chloride ([AMIm][Cl]) and the formation of the regenerated cellulose materials was studied [26]. Recently, ionic liquids are used as swelling pretreatment method followed by hydrolysis of the amorphous cellulose in acid solution. The remarkable swelling and dissolution capacities of ILs render it a promising reaction media for selectively dissolving amorphous regions while preserving crystalline regions for CNCs preparation. It is suggested that the controlled swelling of cellulosic materials in 1-butyl-3-methylimidazolium chloride ([BMIm][Cl]) efficiently prompt the hydrolysis of the amorphous areas while maintaining the crystalline regions. Lorenz et al. reported a two-stage CNCs extraction method involving swelling with [BMIm][Cl] at 80 °C followed by dilute sulfuric acid hydrolysis under mild acid conditions [27]. Fu et al. reported the preparation of thermally stable and surface-functionalized CNCs through a recyclable organic acid and [BMIm][Cl] assisted process under mild conditions [28]. Other kinds of ILs were used to extract CNCs in some other researches. Mao et al. reported a two-step hydrolysis method using [BMIm][HSO_4_] for the extraction of CNCs from common cellulosic materials, which resulted in a high yield of up to 76%, with the sulfur content on the surface of the CNCs remaining at 0.02–0.21% [29]. Abushammala et al. reported the direct extraction of CNCs from wood using 1-ethyl-3-methyl-3-imidazolium acetate ([EMIm][OAc]) treatment [30]. The [EMIm][OAc] with basic character has a strong capability on cellulose dissolution, where anion can form strong H-bond with hydroxyls in cellulose. Previously, solid cation exchange resin has been reported to hydrolysis cellulose to obtain CNCs with a yield of 50% for its recyclability. However, the limited contact between solid acid and cellulose significantly increases the hydrolysis time. Liu et al. successfully prepared CNCs from bleached hardwood pulp by the concentrated phosphotungstic acid, but its reaction time reached as long as 30 h [31].

In this paper, a mild approach was proposed to produce thermally stable cellulose nanocrystals with short time swelling in ionic liquids ([AMIm][Cl]) and hydrolyzed by solid acid (Amberlyst-45). The cellulose materials were swollen in [AMIm][Cl] and subsequently hydrolyzed using Amberlyst-45 ion-exchange resin, leading to a relatively thermostable with no sulfur residues on the products surface. The obtained CNCs were characterized by X-ray diffraction (XRD), Fourier-transform infrared spectroscopy (FTIR), atomic force microscopy (AFM), and thermogravimetric analysis (TGA). The recyclability and reuse of solid acid were investigated, which demonstrated the potential of developing an environmentally friendly, cost-effective, and recyclable process.

## 2. Results and Discussion

### 2.1. Study on ILs Pretreatment

#### 2.1.1. Effects of Different Ionic Liquids and Treatment Temperatures on Fiber Swelling

**Figure 1:**
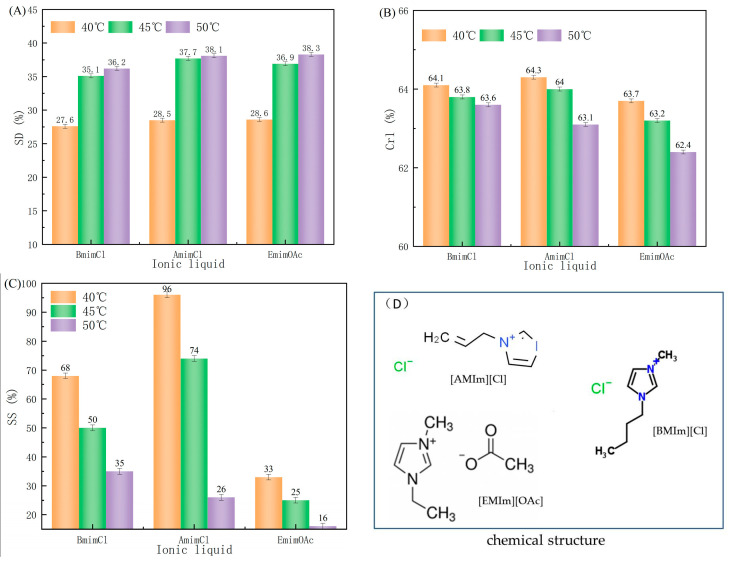
Effects of different species of ionic liquids on (**A**) swelling degree, (**B**) crystallinity degree, and (**C**) swelling selectivity of MCC. (**D**) Chemical structures of the three ionic liquids.

Ionic liquids (ILs) have been gaining increasing attention due to their wide range of advantageous properties such as their compatibility with cellulose materials and their easy recyclability, making them an attractive green solvent. Three types of ILs, [BMIm][Cl], [AMIm][Cl] and [EMIm][OAc], were employed to treat MCC through the disruption of hydrogen-bonding between the anions and hydroxyl groups of cellulose molecules, thus reducing the cohesion of the cellulose molecules. Swelling effects of different treatment temperatures were studied (Figure 1). Figure 1A showed that swelling degrees of [AMIm][Cl] and [EMIm][OAc] treated MCC was higher than that treated with [BMIm][Cl]. The swelling degrees of all samples increased rapidly as the temperature rose from 40 °C to 45 °C and slowed down when the temperature rose to 50 °C. The hydrolysis of hydrogen bonds in cellulose was improved by elevating the temperature, which enhanced its swelling capability by facilitating the disruption of intramolecular and intermolecular hydrogen bonds.

Cellulose is composed of both crystalline and amorphous regions. The crystalline region of cellulose has an ordered and compact structure, with low reactivity and accessibility. On the other hand, the amorphous region of cellulose has a loose arrangement of cellulose chains and is characterized by high accessibility and reactivity. During IL treatment, the oxygen and hydrogen atoms of cellulose formed electron donor–electron acceptor (EDA) complexes with the charged species of the IL, primarily between the C-6 and C-3 hydroxyl groups of neighboring cellulose chains. The interaction between the IL and cellulose led to the separation of the hydrogen bonding between the cellulose chains, resulting in the segregation of the cellulose chains in the IL. This segregation preferentially occurs in the amorphous region of the cellulose due to its higher accessibility compared to the crystalline region, allowing the IL to attack the amorphous region at mild conditions while leaving the crystalline region intact. The different crystallinity degrees observed in Figure 1B indicated that the type of ionic liquid and the treatment temperature had varied impacts on the crystallinity degree. The effect of [EMIm][OAc] on the crystalline region of cellulose was more pronounced and the degree of crystallinity was significantly decreased. The degree of crystallinity was also affected by the treatment temperature. As the treatment temperature increased, the chemical action strengthened and the degree of crystallinity decreased, especially when the temperature reached 50 °C.

In addition to swelling degree (SD), swelling selectivity (SS) was also used to evaluate the swelling effect. Figure 1C revealed that the swelling selectivity of MCC treated with [AMIm][Cl] was higher than that of those treated with [EMIm][OAc], leading to an improved hydrolysis reaction in the second step and an increased yield and crystallinity of the resulted CNCs. In conclusion, the highest treatment efficacy was achieved with the use of [AMIm][Cl] ionic liquid at 45 °C. The use of [AMIm][Cl] had been demonstrated to be an effective way to break the hydrogen bonds in the amorphous region of cellulose, thus increasing its reactivity and accessibility, while also being cost-effective to produce, making it an appropriate solvent for cellulose swelling.

#### 2.1.2. Images of MCC at Different Swelling Times in [AMIm][Cl]

**Figure 2:**
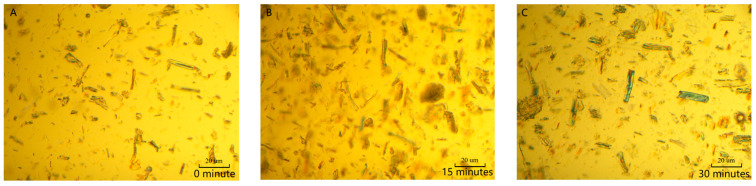

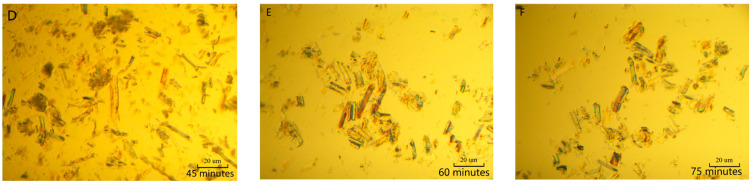
Effect of swelling time on sizes of MCC in [AMIm][Cl]. ((**A**) 0 min; (**B**) 15 min; (**C**) 30 min; (**D**) 45 min; (**E**) 60 min; (**F**) 75 min).

The morphology of MCC was observed by polarizing microscope (Jiangnan Yongxin NP-800RF/TRF, Ningbo, China) after being swollen in the [AMIm][Cl] for varying residence times. The weakened hydrogen-bonding network of the amorphous region of cellulose made it more susceptible to attack by [AMIm][Cl], resulting in an increase in its size. Polarizing microscopy analysis indicated that the width of MCC increased with the extension of swelling time (Figure 2).

#### 2.1.3. Evaluation of Pretreatment Conditions of MCC in [AMIm][Cl]

**Figure 3:**
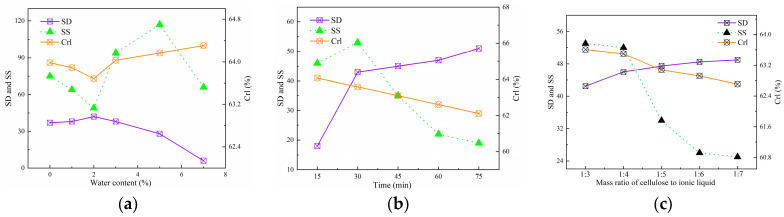
Effects of swelling parameters on the properties of the MCC in [AMIm][Cl]. (**a**) Effect of water content. (**b**) Effect of time. (**c**) Effect of mass ratio of cellulose to ionic liquid.

The effects of pretreatment time, water content, and mass ratio of cellulose to ionic liquid of the system were investigated (Figure 3). Cellulose fibers are difficult to swell in complete anhydrous condition in industrial production, since both ionic liquids and cellulose have water absorption ability offered by their strong hydrophilic group. The swelling effects of water content in the pretreatment system were investigated under the conditions of a 1:3 mass ratio of cellulose to ionic liquid, a reaction temperature of 45 °C and a reaction time of 30 min. As the water content increased up to 2%, the swelling degree gradually increased until reaching its peak value, then decreased sharply, and almost no swelling effect was observed when the water content exceeded 7% (Figure 3a). The effectiveness of ionic liquids in disrupting hydrogen bonds with cellulose was reduced as the water content increased due to the anions of the ionic liquids forming hydrogen bonds with water molecules in the system. The [AMIm][Cl] mainly acted on the amorphous region of cellulose, which is characterized by a loose structure and high accessibility in the swelling treatment. The addition of a small amount of water increased the specific activity of [AMIm][Cl] on the amorphous region by promoting the formation of larger-volume hydrated anions, resulting in an effective swelling effect. It can be observed that the crystallinity of cellulose decreased first and then increased with the increase of water content. A small amount of water molecules could effectively hydrate the amorphous region of cellulose, providing a channel for ionic liquid to act on the crystalline region, thus leading to a slight decrease in the crystallinity of cellulose. When the moisture content exceeded 2%, the hydrogen bonding ability of the ionic liquid was significantly reduced, and the crystallinity of cellulose increased after swelling.

The swelling effects of pretreatment time were studied under the conditions of reaction temperature of 45 °C, mass ratio of cellulose to ionic liquid 1:3, and water content of 2.0% (Figure 3b). The swelling degree showed a rapid increase with the increment of treatment time, and then the trend slowed down when the treatment time surpassed 30 min. As the treatment time increased, the swelling degree of the cellulose showed an initial increase followed by a decrease, reaching a peak at 30 min. The decrease in the crystallinity index with increasing time indicated the disruption of the crystalline regions. The initially slight and then rapid change in the crystallinity index was due to the preferential attack of [AMIm][Cl] on different regions during the swelling process. The swelling agent initially acted on the amorphous regions, resulting in a decrease of the crystallinity index and a sharp decrease in the selectivity of swelling after 30 min, when the amorphous regions were fully swollen while the crystalline regions were preserved intact.

The swelling effects of mass ratio of cellulose to ionic liquid of the system were studied under conditions of reaction temperature 45 °C, reaction time 30 min, and water content 2.0% (Figure 3c). Figure 3c showed that the swelling degree of cellulose increased with the increasing mass ratio of cellulose to ionic liquid, from 1:3 to 1:7; however, the rate of increase diminished when the ratio reached 1:4. When the cellulose content was high, the inadequate anions provided by the solvent system resulted in poor swelling. The crystallinity index and selectivity of swelling decreased with the increase of the mass ratio of cellulose to ionic liquid, indicating that more of the active groups of the ionic liquid were able to interact with the crystalline region of the cellulose. The optimal mass ratio of cellulose to ionic liquid was 1:4, which provided the best compromise between SD, SS, reduced consumption of ionic liquid, cost-effectiveness of recovery, and facile blending of the cellulosic fibers and ionic liquid. Based on the results, the optimal swelling conditions adopted were 2% water content in [AMIm][Cl], 30 min treatment time, and 1:4 mass ratio of cellulose to ionic liquid.

### 2.2. Morphology of CNCs

**Figure 4:**
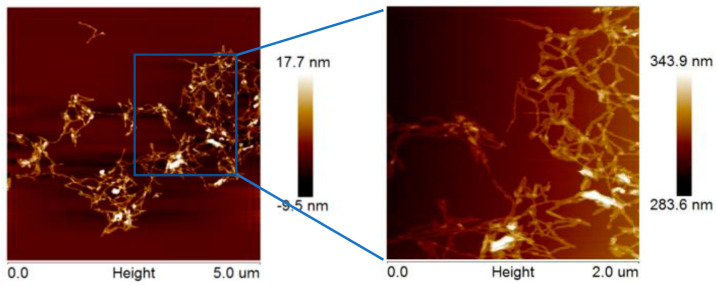
AFM images of CNCs extracted via swelling−SA−catalyzed hydrolysis process.

Photographs of the suspensions indicated that the CNCs were in a pale milky white colloidal suspension with no visible aggregation or sedimentation observed after a three-month period at room temperature. The AFM images in Figure 4 revealed the uniform size distribution of the obtained CNCs with rodlike morphology, with an average length of 300 ± 100 nm and a mean diameter of 20 ± 10 nm, as determined by NanoScope Analysis software. The results indicated that CNCs of similar size to the commercial nanocellulose were successfully prepared.

### 2.3. Characterization of CNCs Structure

#### 2.3.1. X-ray Diffraction Analysis

**Figure 5:**
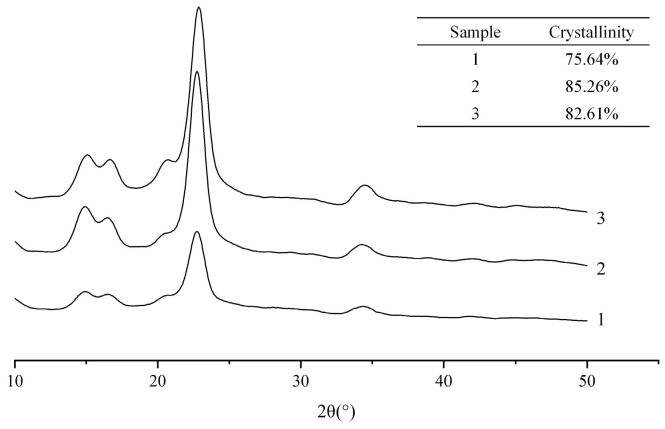
X-ray spectra of (1) the original MCC, (2) the CNCs extracted with swelling−SA−catalyzed hydrolysis method at 45 °C for 5 h, and (3) the extracted CNCs hydrolyzed in 65.0 wt% H_2_SO_4_ at 45 °C for 5 h.

The crystalline structure of MCC and the CNCs extracted by the two different methods were analyzed using X-ray diffraction (XRD) (Figure 5). It was showed that the intense peaks at around 2θ = 14.9° (1$\bar{1}$0), 16.4° (110), 22.8° (200) and 34.5° (004) for cellulose I CNCs kept their characteristics after the swelling and hydrolysis process in [AMIm][Cl], implying that the pretreatment mainly acted on the amorphous region. The crystallinity indexes of the original MCC, solid acid extracted CNCs, and sulfuric acid extracted CNCs were calculated to be 75.64%, 85.26% and 82.61%, respectively, suggesting a slight increase in the relative intensities of the crystalline and amorphous peaks. When MCC was swollen in [AMIm][Cl], the improved accessibility and reactivity of the amorphous region during subsequent solid acid hydrolysis compared to the traditional sulfuric acid method was observed. Solid acid catalyzed hydrolysis showed superior performance over sulfuric acid in selectively hydrolyzing the amorphous regions of cellulose while retaining the crystalline parts. This led to a higher crystallinity index of the products.

#### 2.3.2. FTIR Characterization

**Figure 6:**
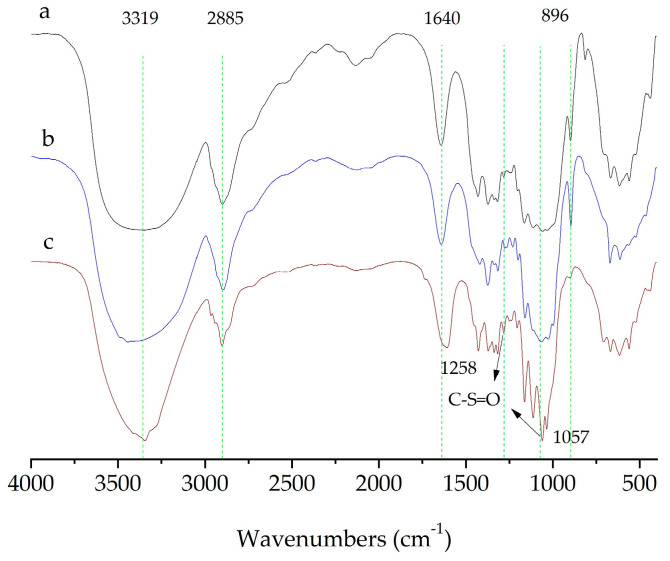
FTIR spectra of (**a**) the original MCC; (**b**) the CNCs extracted with swelling−SA−catalyzed hydrolysis method for 5 h at 45 °C; (**c**) the extracted CNCs hydrolyzed in 65.0 wt% H_2_SO_4_ for 5 h at 45 °C.

The structural properties of CNCs prepared with two different methods and the original MCC was characterized by analyzing their FTIR spectra. FTIR spectra of the cellulose sample (Figure 6) showed absorption bands related to the cellulose structure, including the hydroxyl stretching vibration at 3319 cm^−1^, the C-H vibration at 2885 cm^−1^, and vibrations of β-glycosidic linkages at 896 cm^−1^. Additionally, a peak at 1640 cm^−1^ indicated the presence of a small amount of moisture in the sample.

Two special weak absorption bands at 1057 cm^−1^ and 1258 cm^−1^ were found on CNCs extracted with traditional methods, indicating the presence of sulfonic acid groups, which were produced by the esterification of hydroxyl groups on cellulose and sulfuric acid. However, the CNCs isolated with swelling-SA-catalyzed hydrolysis method keep a typical spectrum of cellulose structure, and no characteristic absorption of sulfonic acid was observed in the product.

### 2.4. Properties of CNCs

#### 2.4.1. Size Distribution of CNCs

**Figure 7:**
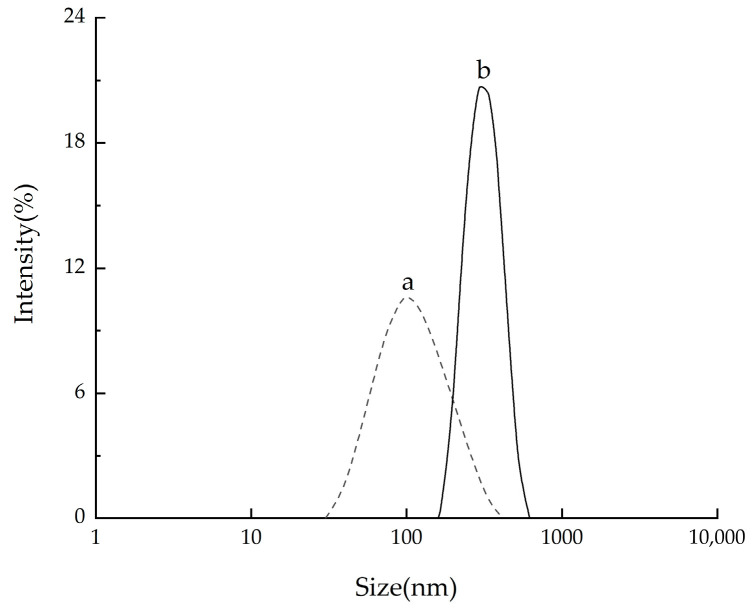
Size distribution of CNCs with different methods. a: the extracted CNCs hydrolyzed in 65.0 wt% H_2_SO_4_ at 45 °C for 5 h; b: the CNCs extracted with swelling−SA−catalyzed hydrolysis method at 45 °C for 5 h.

The particle size distributions of CNCs extracted by the two methods were analyzed by the laser diffraction particle size analyzer (Zetasizer Nano ZS90, Malvern Panalytical Ltd., Malvern, UK) (Figure 7). The average lengths of CNCs extracted by the two methods were found to be 467 nm and 178 nm, respectively, which were within the nanoscale range. The swelling-SA-catalyzed hydrolysis method resulted in a narrower and more uniform length distribution of CNCs compared to traditional hydrolysis, which was attributed to its more uniform reaction.

#### 2.4.2. Thermostability of CNCs

**Figure 8:**
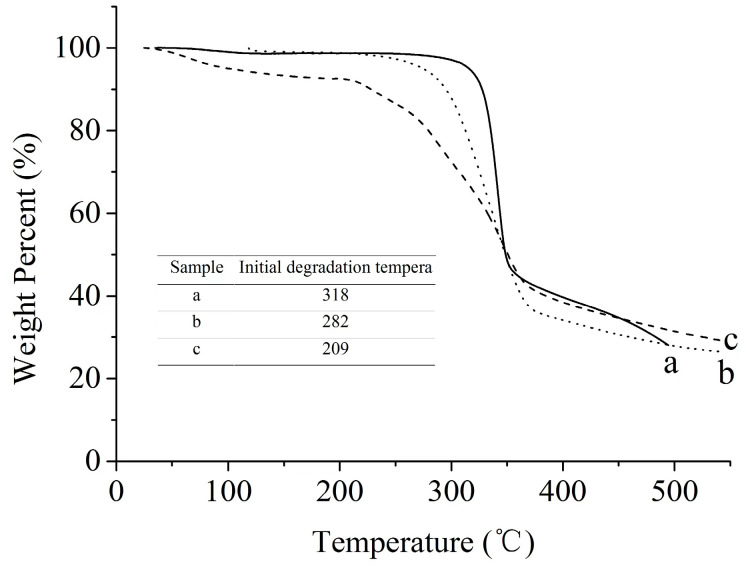
TGA analysis of a: the original MCC, b: the CNCs extracted with swelling−SA−catalyzed hydrolysis method at 45 °C for 5 h, and c: the extracted CNCs hydrolyzed in 65.0 wt% H_2_SO_4_ at 45 °C for 5 h.

The thermostability of CNCs prepared using different methods was evaluated on a thermogravimetric analyzer (Figure 8). Conventional sulfuric acid hydrolysis procedure eliminated the amorphous regions of MCC and simultaneously resulted in the esterification of sulfuric acid to the hydroxyl groups on the surface of the extracted CNCs, thus reducing the thermal stability of the CNCs. At temperatures below 200 °C, the weight loss was mainly attributed to water evaporation from the samples, as reported previously [29]. Due to its high hydrophilicity, CNCs prepared by conventional method exhibited higher weight loss in the atmospheric humidity absorption stage than those prepared by swelling-SA-catalyzed hydrolysis method. When the temperature was above 200 °C, the CNCs extracted by the swelling-SA-catalyzed hydrolysis method exhibited significantly improved thermosability compared to those extracted by sulfuric acid hydrolysis. Figure 8 showed that the initial decomposition temperature (282 °C) of the swelling-SA-catalyzed hydrolysis extracted CNCs was significantly higher than that of sulfuric acid hydrolysis extracted CNCs (209 °C) but slightly lower than that of the original MCC (318 °C). The enhanced thermal performance of swelling-SA-catalyzed hydrolysis extracted CNCs was attributed to two factors: (1) selective swelling and high crystallinity of the CNCs and (2) the absence of sulfonic acid groups in the hydrolyzed CNCs, which improved its thermal stability. The results of the swelling-SA-catalyzed hydrolysis method for CNCs preparation confirmed that the weight loss of the prepared CNCs was nearly the same as that of the original cellulose, indicating that almost no sulfate groups were present on the CNCs. The high thermal properties of the CNCs could further expand its application scope in cellulose-based biocomposites processing, especially at elevated temperatures.

### 2.5. The Recyclability of the Solid Acid

**Figure 9:**
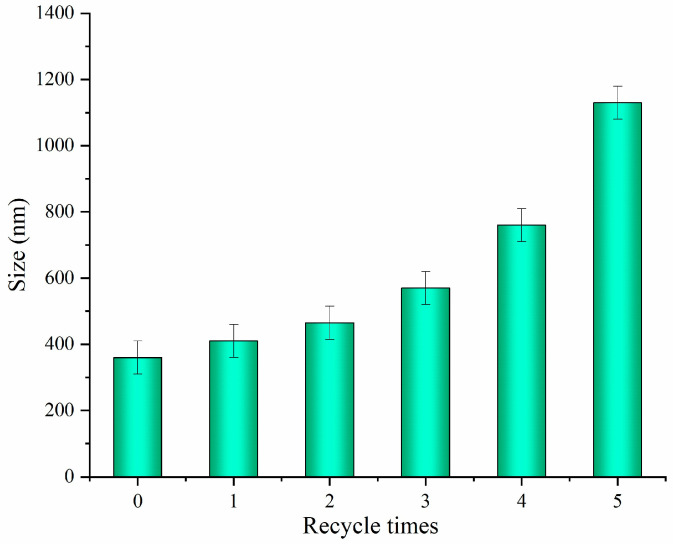
Length of the CNCs after each reuse cycle.

Considering the environmental protection and technical application, the recycling of acid is very important. Figure 9 showed that the recycled solid acid was reused for the extraction of CNCs up to five times via the same acidolysis process, with the lengths of the extracted CNCs measured each time. The results indicated that the length of CNCs increased progressively with the increasing number of recycles, suggesting a decrease in the acidolysis ability of the solid acid upon reuse. After the fourth and fifth cycle of reuse, the solid acid exhibited a significant decrease in acidity, resulting in an increase in the length of CNCs to over 800 nm and 1000 nm, respectively, exceeding the nanocellulose category. The decreased hydrolytic activity of recycled solid acid may be due to the adsorption of fiber-degrading components on its surface active sites during the recycling process. Ultrasonic conditioning was applied for surface cleaning to restore the hydrolytic activity of the solid acid, resulting in a reduction of the CNCs length to 720 nm after the fifth recycling cycle. Solid acids could be recycled up to three times without the surface cleaning.

## 3. Materials and Methods

### 3.1. Materials

Microcrystalline cellulose (MCC) with degree of polymerization (DP) of 175 was purchased from Anhui Shanhe Pharmaceutical. Co., Ltd. (Huainan, China). 1-butyl-3-methylimidazolium chloride ([BMIm][Cl]), 1-allyl-3-methylimidazolium chloride ([AMIm][Cl]) and 1-ethyl-3-methyl-3-imidazolium acetate ([EMIm][OAc]) with purity of 99.0% were obtained from J&K Scientific LLC (San Jose, CA, USA). Solid acid (Amberlyst-45 ion-exchange resin) was supplied by Nanda Synthetic Chemistry Co., Ltd. (Jiangyin, China).

### 3.2. Nanocrystal Extraction

Before the experiment, MCC was vacuum-dried at 60 °C for 24 h, and ionic liquids were oven-dried at 105 °C for 12 h in order to completely remove the moisture content. Cellulose nanocrystals extraction was carried out using ILs ([BMIm][Cl], [AMIm][Cl] and [EMIm][OAc]) and ion-exchange resin (Amberlyst-45) via two steps involving swelling and hydrolysis. Swelling was occurred in IL (mass ratio of cellulose to ionic liquid 1:3 to 1:7) with different quantities of water content from 0 to 7 wt% in a flask at 40 to 50 °C for 15 to 75 min, assisted with ultrasonic treatment in 2103QTD Ultrasonic Generator (Kunshan Ultrasonic Instrument Co., Ltd., Kunshan, China) with frequency of 40 kHz and power of 360 W. In the second step, a predetermined volume of water and Amberlyst-45 were added to the mixture and a 45 wt% solid acid (relative to cellulose) was employed to extract CNCs via a 5.0-h hydrolysis process at 45 °C.

After the reaction was complete, deionized water was added to the mixture and the solid acid was recycled by filtration. The MCC that had not been hydrolyzed was removed by centrifugation (C160048R, Xiangyi centrifuge instrument Co., Ltd., Changsha, China) at a speed of 13,000 r/min. The CNCs were further purified by dialysis to remove residual ionic liquid and glucose.

### 3.3. Microscopy and Calculation of Swelling Degree of MCCs

The morphology and width of MCC during the swelling step was directly observed and calculated using NP-800RF/TRF Polarizing Microscope (Nanjing Jiangnan Novel Optics Co., Ltd., Nanjing, China). *SD* (swelling degree) of MCC was calculated according to Equation (1).

(1) $$SD\left(\%\right)=\frac{W_{t}-W_{o}}{W_{o}}\times 100\%$$

where *W_o_* is the width (μm) of the original MCC, and *W_t_* is the width (μm) of the swelling treated fibers.

The selectivity of swelling (*SS*), which represents the efficiency of swelling treatment, was also calculated according to Equation (2).

(2) $$SS=\frac{SD\times Y}{DCrI}$$

where *SD* is the swelling degree (%), *D_CrI_* is the decrease of the degree of crystallinity (%), and *Y* is the swelling yield (%).

### 3.4. X-ray Diffraction (XRD) Analysis and Determination of Crystallinity Index

The crystallinity index and crystalline structure of MCC and extracted CNCs were obtained using X-ray diffractometer (D8 Advance, Bruker, Karlsruhe, Germany), within a scanning angle range of 10–50° and a scanning speed of 0.18° min^−1^. The obtained CNCs by the two methods were freeze-dried separately before analysis. The relative crystallinity index (*CrI*) was calculated using the following empirical Equation (3).

(3) $$CrI\left(\%\right)=\frac{I_{200}-I_{am}}{I_{200}}\times 100\%$$

where *I*_200_ is the peak intensity of the main crystalline plane (200) diffraction at 22.8°, and Iam is the diffraction intensity of the amorphous domain of cellulose at 18.0°.

### 3.5. Particle Size Distribution

The particle size distribution of the extracted CNCs was measured using Zetasizer Nano ZS instrument (Malvern Panalytical Ltd., Malvern, UK) at room temperature. The sample suspensions with a concentration range from 0.1–40 ppm were pretreated ultrasonically using a 2103QTD Ultrasonic Generator (Kunshan Ultrasonic Instrument Co., Ltd., Kunshan, China) with a frequency of 40 kHz and a power of 360 W for 20 min before analysis. Each sample was measured in parallel for three times, and the average data was reported.

### 3.6. FT-IR Analysis of the Original MCC and CNCs

FT-IR analysis was performed with an alpha FT-IR spectroscopy (VERTEX 70, Bruker, Bremen, Germany) to analyze the functional groups of the original MCC and extracted CNCs samples. The obtained CNCs by the two methods were freeze-dried separately before analysis. The spectra were measured in ATR mode and recorded in absorbance mode within wavenumber from 4000 to 400 cm^−1^ with a nominal resolution of 2 cm^−1^.

### 3.7. Atomic Force Microscope (AFM) Observation

AFM observation of the extracted CNCs was performed with a Mutimode 8 (Bruker, San Jose, CA, USA). An ultrasonically treated CNCs slurry (with a concentration of 0.001 wt%) was dropped onto a freshly mica substrate and left to dry for 20 min before analysis.

### 3.8. Thermogravimetric (TG) Analysis

The TG analysis on CNCs samples was carried out using the Simultaneous Thermal Analyzer Q50 (New Castle, DE, USA) instrument with a heating rate of 10 °C/min from 30 to 500 °C under nitrogen atmosphere.

## 4. Conclusions

A swelling-SA-catalyzed hydrolysis method was developed for the extraction of CNCs from MCC, which involves a two-step procedure of swelling and hydrolysis. The optimum conditions for swelling were determined to be 2 wt% water content, 30 min treatment time, a 1:4 mass ratio of cellulose to [AMIm][Cl] and 45°C. In the second step, CNCs were extracted by solid acid hydrolysis at 45 °C for 5.0 h with a solid acid amount of 45 wt% based on cellulose. The obtained CNCs had high thermal stability and rodlike morphology, with an average length and width of 300 ± 100 nm and 20 ± 10 nm, respectively. Additionally, the solid acid could be reused up to three times without surface purification.

## Data Availability

Data is available on request due to privacy restrictions.

## Sample Availability

Samples of the compounds are not available from the authors.
